# Mapping feature-sensitivity and attentional modulation in human auditory cortex with functional magnetic resonance imaging

**DOI:** 10.1111/j.1460-9568.2011.07656.x

**Published:** 2011-05

**Authors:** Aspasia E Paltoglou, Christian J Sumner, Deborah A Hall

**Affiliations:** 1 MRC Institute of Hearing Research, University ParkNottingham, UK; 2 Division of Psychology, School of Social Sciences, Nottingham Trent UniversityBurton Street, Nottingham NG1 4BU, UK

**Keywords:** auditory motion, frequency modulation, localizer contrast, selective attention

## Abstract

Feature-specific enhancement refers to the process by which selectively attending to a particular stimulus feature specifically increases the response in the same region of the brain that codes that stimulus property. Whereas there are many demonstrations of this mechanism in the visual system, the evidence is less clear in the auditory system. The present functional magnetic resonance imaging (fMRI) study examined this process for two complex sound features, namely frequency modulation (FM) and spatial motion. The experimental design enabled us to investigate whether selectively attending to FM and spatial motion enhanced activity in those auditory cortical areas that were sensitive to the two features. To control for attentional effort, the difficulty of the target-detection tasks was matched as closely as possible within listeners. Locations of FM-related and motion-related activation were broadly compatible with previous research. The results also confirmed a general enhancement across the auditory cortex when either feature was being attended to, as compared with passive listening. The feature-specific effects of selective attention revealed the novel finding of enhancement for the nonspatial (FM) feature, but not for the spatial (motion) feature. However, attention to spatial features also recruited several areas outside the auditory cortex. Further analyses led us to conclude that feature-specific effects of selective attention are not statistically robust, and appear to be sensitive to the choice of fMRI experimental design and localizer contrast.

## Introduction

Selective attention improves sensory perception by enhancing the neural representation of the stimulus or feature of interest and/or by attenuating the representation of competing stimuli (Tootell *et al.*, 1998; Treue & Trujillo, 1999). In the visual system, localized increases in activity have been reported in many different cortical regions, when attention is directed to the feature that is processed within that region (e.g. Corbetta *et al.*, 1990). In the auditory system, clear demonstrations of feature-specific attentional enhancement have been less ubiquitous (Petkov *et al.*, 2004; Johnson & Zatorre, 2005, 2006; Degerman *et al.*, 2006; Paltoglou *et al.*, 2009). Three recent functional magnetic resonance imaging (fMRI) studies demonstrated enhancement for spatial, but not for nonspatial, features (Ahveninen *et al.*, 2006; Krumbholz *et al.*, 2007; Altmann *et al.*, 2008). Thus, the extent and nature of auditory attention-related enhancement remain unclear.

One important issue is that task difficulty can influence attentional modulation (Boudreau *et al.*, 2006; Atiani *et al.*, 2009). For all three studies mentioned above, there was some indication that the spatial task was more difficult than the nonspatial task. It may be crucial to equate performance across attentional conditions.

Another important issue in such studies is the method used to define the feature-sensitive region within which the response to attention is measured. Krumbholz *et al.* (2007) mapped the pitch-sensitive and location-sensitive regions by requiring subjects to attend to an irrelevant auditory feature. They reasoned that controlling for attention may better eliminate the effects of higher-order cognition on sensory activity, and thus define a brain region that is more feature-specific than one defined during passive listening. This approach still assumes the logic of pure insertion (i.e. that only the feature of interest differs across the two conditions) (Price & Friston, 1997), and recent auditory fMRI evidence shows that this is not always correct (Garcia *et al.*, 2010). Interactions between feature-sensitive coding and the listening context could also be attributed to the main effect of feature-sensitive coding. Furthermore, arguments have been advanced against the practice of using the same stimulus conditions (or subset thereof) for localization and the effect of interest, because it introduces the risk of making invalid statistical inferences whenever the results are not inherently independent of the selection criteria (Kriegeskorte *et al.*, 2009). One way to ensure independence is to choose different pairs of conditions for defining the region of interest and for assessing the effect of selective attention.

The present study examined the neural correlates of feature-specific auditory selective attention to frequency modulation (FM) and auditory motion, which engage distinct auditory cortical regions (Hart *et al.*, 2004). The difficulty of the tasks was matched as closely as possible by adjusting the stimulus parameters for each participant in ‘pre-scanning’ sessions. The effect of attention was examined by considering the magnitude of activity across conditions within the separate regions of the auditory cortex that were sensitive to motion and to FM. The experimental design allowed us to evaluate how the choice of localizer might affect the conclusions drawn from the data.

## Materials and methods

### Participants

Sixteen participants with normal hearing (≤ 25-dB hearing level in octave steps from 250 to 8000 Hz) and normal (or corrected to normal) vision completed the performance-matching and fMRI phases of the experiment. There were seven males and nine females, with a mean age of 24.7 years (range, 18–37 years). All but one of the participants were right-handed. None of the participants had any history of neurological problems. Participation in the fMRI experiment required performance to reach a percentage measure of the hit rate minus the false-alarm (FA) rate of 60–90%, with a no more than 5% difference in performance across the two target-detection tasks. A further 15 participants were screened, but were excluded from the fMRI experiment because performance matching did not meet the criteria. Their data are not reported here. All experimental procedures were approved by the University of Nottingham Medical School Research Ethics Committee, and participants gave written informed consent.

### Pre-scanning assessment

In order to match detection performance for the frequency-modulated and spatial targets, the parameters defining both targets were modified individually on the basis of pre-scanning assessments performed in a sound-proofed booth. Parameters were chosen so that performance in detecting the frequency-modulated and spatial targets fell within the range 60–90%, and also so that the two measures of hit rate and FA rate did not differ by more than 5% within any individual participant.

#### Condition

Performance matching was carried out with a reference condition in which the sound was a moving frequency-modulated signal (denoted Mo + FM).

#### Stimuli

**Fig. 1:**
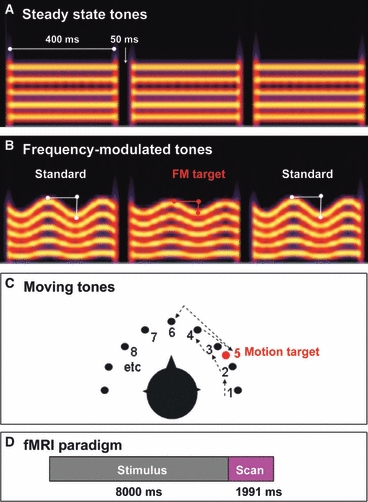
(A) Illustration showing three of 16 tones of the static stimulus sequence. The five horizontal lines illustrate the fundamental (lower line) and the four harmonics. (B) Illustration showing three of 16 tones of the frequency-modulated stimulus sequence. The middle tone is the target. (C) Illustration of the moving stimulus, bird's-eye view. The numbers and the dotted arrows depict the order of appearance of the tones. The red circle illustrates the target (the fifth tone in this particular example). (D) fMRI paradigm. Each stimulus block lasted for 8 s. As soon as the 8-s stimulus ended, image acquisition was initiated.

The stimuli were complexes of 18 simultaneous, harmonically related tones (fundamental frequency, 400 Hz; harmonics numbered 1–5; 400-ms duration; 10-ms onset and offset ramps, 50-ms inter-stimulus interval). The perceived spatial location and spectral modulation of the complex tones were manipulated in the following way. The FM reference had a modulation rate of 5 Hz and a modulation depth of 12.5% of the fundamental frequency (i.e. 50 Hz). As Fig. 1B shows, the modulation occurred within each tone.

For the moving reference, the location was fixed for each individual stimulus, but successive stimuli shifted in their interaural time difference (ITD), in 150-μs steps. This gave the percept of a sound source sweeping horizontally from one side to the other and then back again. For half of the moving sequences, the sound source started on the left (ITD, −600 μs), and for the others it started on the right (ITD, +600 μs). Each reference sequence contained one or two targets that were defined either by a change in the expected modulation depth or a change in the expected direction of motion (Fig. 1B and C). The number of targets in each sequence was fully randomized across conditions. Frequency-modulated targets were defined by a shallower modulation depth, whereas motion targets were defined by a perceived jump in the opposite direction to the primary arc of motion. The frequency-modulated target was initially defined by 8.5% modulation depth, and the motion target was initially defined by a 350-μs shift, both of which were clearly perceptible by all participants. Targets could occur at any temporal position in the sequence, except for the first two or last two tones.

#### Task

Stimuli were presented through Sennheiser (type HD480II) headphones at 87 dB SPL. At the start of each sequence, a visual instruction indicated whether participants should detect the FM or the motion targets. The instruction remained on the computer screen throughout the sequence. Each assessment run comprised 52 sequences of FM detection and 52 sequences of motion detection, with the instructions appearing in a randomized order. For all conditions, participants had to press a button as soon as they heard the oddball (target) tone.

Target-detection performance was computed by use of a percentage measure of the hit rate minus the FA rate. A hit was scored for a response within a 1200-ms time window following target onset. A false alarm was scored for a response at any other time point. Depending on individual performance, target parameters were modified for the subsequent stage of the pre-scanning assessment. Up to four runs were required to reach the necessary performance criteria. Across participants, chosen target parameters ranged from a 100 to a 350-μs jump in the azimuthal direction, and from an 8.75% to an 11.5% target modulation depth (Table 1).

**Table 1:** Performance matching across the attention-demanding conditions

	Chosen target parameters		fMRI performance			
		
Participant	Spatial target (ITD, μs)	Frequency-modulated target (depth, %)	aMO (Mo + FM)	aFM (Mo + FM)	aMO (Mo + nonFM)	aFM (Stat + FM)
01	150	11.00	83	87	93	89
02	300	9.35	57	82	79	73
03	200	11.50	89	93	81	95
04	150	10.87	80	92	85	93
05	350	8.75	59	75	73	82
06	350	10.50	83	79	82	65
07	150	10.50	83	83	93	83
08	150	11.00	90	80	94	90
09	200	11.00	67	76	80	77
10	150	10.00	91	95	87	93
11	250	9.50	96	98	96	93
12	300	11.50	97	95	97	95
13	350	10.00	95	91	100	100
14	200	10.00	74	79	72	75
15	300	10.80	81	80	87	80
16	200	11.50	80	83	76	80
Mean	234.4	10.49	81.6	85.5	85.9	85.2

Performance is reported as a percentage measure of the hit rate minus FA rate. Hit rate is defined by no. of hits\(no. of hits + no. of misses), and FA rate is defined by no. of false alarms\(no. of false alarms + no. of correct rejections).

### fMRI experiment

The fMRI study was a factorial design that partially crossed three tone sequences with three listening instructions to generate seven sound conditions.

#### Conditions

The three tone sequences contained different combinations of FM and spatial motion: (i) moving FM (Mo + FM); (ii) stationary FM (Stat + FM); and (iii) moving steady state with no FM (Mo + nonFM). The three listening tasks were: (i) passive listening (prefixed ‘p’); (ii) attend motion (prefixed ‘aMo’); and (iii) attend FM (prefixed ‘aFM’). The resulting seven conditions were three passive listening conditions [p(Mo + FM), p(Mo + nonFM), and p(Stat + FM)], two attend motion conditions [aMo(Mo + FM) and aMo(Mo + nonFM)], and two attend FM conditions [aFM(Mo + FM) and aFM(Stat + FM)]. An additional silent condition was included as a baseline.

#### Stimuli

The Mo + FM sequences were identical to those used in the pre-scanning assessment. Similar reference parameters were used to create the Stat + FM and Mo + nonFM sequences, although the Stat + FM sequence had a fixed ITD of 0 μs, and the Mo + nonFM sequence had a zero modulation depth. All targets were defined individually according to performance on the pre-scanning assessment. However, the three passive listening conditions contained no targets.

#### Task

Stimuli were presented with custom-made magnetic resonance-compatible headphones at a level of 95 dB SPL. Task instructions (‘X’, ‘attend FM’, or ‘attend motion’) were projected onto a screen placed at the foot of the scanner bed, and viewed with a pair of angled mirrors mounted on the head coil. Instructions subtended a visual angle of 4° and were clearly legible for all participants. The ‘X’ denoted passive listening, whereby participants were told to listen carefully. Target-detection responses were made with a right-handed button press that was recorded for off-line analysis. Conditions were presented in a pseudo-randomized order that changed after every pair of sequences. The order was counterbalanced across participants. In total, the fMRI experiment comprised 26 different 8-s sequences for each sound condition, plus 30 8-s epochs for the silent condition.

### fMRI protocol

Scanning was performed on a Philips 3 Tesla Intera MR scanner (Achieva/Intera Release 1.2/11) equipped with an eight-channel SENSE head coil. Scanning took place at the Sir Peter Mansfield Magnetic Resonance Imaging Centre, University of Nottingham.

For each participant, a T1-weighted anatomical scan was acquired to facilitate processing of the functional image data. The anatomical scan was composed of 160 sagittal slices at 1-mm^3^ resolution (matrix size, 256 × 256), and was completed in about 5 min. The anatomical scan was used to select the orientation of the functional scans. Each scan consisted of 32 oblique axial slices at 3-mm^3^ resolution (matrix size, 64 × 64; flip angle, 90°; echo time, 36 ms; acquisition time, 1971 ms) to include the whole brain. The lower slice cut across the cerebellum and prefrontal cortex, and the upper slice cut across the superior parietal cortex (leaving out a small part of the parietal cortex, at the top). We used a sparse sampling method (repetition time, 10 s) (Hall *et al.*, 1999) to minimize the temporal overlap between the intense scanner noise and the tone sequence (Fig. 1D). A total of 106 scans were collected in each experimental run. The experiment was divided into two 17-min runs.

### fMRI analysis

Spatial pre-processing and volumetric image analysis was performed with spm2 software (http://www.fil.ion.ucl.ac.uk/spm/). Functional time series were motion-corrected to account for head movements both within and between the two runs, using the last scan of the first run as a reference. Head movements did not exceed 3 mm (translation) and 3° (rotation). First, the anatomical and functional scans were co-registered, and then the anatomical scan was segmented into the different tissue components. Next, the grey matter image was spatially transformed to match a template that represents the *a priori* voxel-wise probabilities of finding grey matter. Finally, the same transformation parameters were applied to the anatomical scan and the fMRI time series. The transformed anatomical scan preserved its voxel resolution of 1 mm^3^. Functional scans were upsampled to 2 mm^3^ and then smoothed by a Gaussian kernel of 8-mm full width at half-maximum.

Individual time series data were analysed within the framework of a general linear model. Individual model specification included 14 regressors: seven describing each of the experimental conditions of interest, six describing the realignment parameters to account for any residual head motion, and one to account for session differences between the two runs. The silent baseline condition was implicitly modelled. Low-frequency artefacts were removed by high-pass filtering the time series. The high-pass filter cut-off was 0.0013–0.0017 Hz, depending on the length of the intervals between repetitions of the same experimental condition. For each participant, the effect of each experimental condition of interest was computed and used as input to group-level random-effects analyses. The main aim of the group analysis was to localize the sensory response to motion and FM using passive listening contrasts [i.e. p(Mo + FM) > p(Stat + FM) and p(Mo + FM) > p(Mo + nonFM), respectively]. For comparison with Krumbholz *et al.* (2007), sensory responses were localized in a secondary group analysis by the use of active listening contrasts [i.e. aFM(Mo + FM) > aFM(Stat + FM) and aMO(Mo + FM) > aMO(Mo + nonFM)]. For clarity, these contrasts are listed in Table 2. Note that the two strategies for localizing activity used the same reference sequence, but differed in the behavioural context. [According to Donders (1868), distinct cognitive activities can be separated by using a subtraction design in which the only feature that distinguishes the two conditions is the cognitive process of interest. Although the assumption of pure insertion has received criticism (e.g. Friston *et al.*, 1996), it nevertheless dominates fMRI methodology and certainly underpinned the design choice in the study by Krumbholz *et al.* (2007). We employ it here with caution, and comment later when we discuss the comparison between passive and active localizers]. Statistical significance was tested by correcting for multiple comparisons within a volume of the superior temporal gyrus that had been hand-drawn using the group-averaged normalized anatomical scan. Corrections used the false discovery rate (FDR) (Genovese *et al.*, 2002) to reduce type II errors. All results exceeding a cluster-level significance of *P* = 0.05 are reported.

**Table 2:** Summary of the three different pairs of statistical contrasts performed at the group level

Type of response	Statistical contrast
Passive localizers	
Motion-sensitive response	p(Mo + FM) > p(Stat + FM)
FM-sensitive response	p(Mo + FM) > p(Mo + nonFM)
Enhancement by selective attention	
Motion	aMo(Mo + FM) > aFM(Mo + FM)
FM	aFM(Mo + FM) > aMo(Mo + FM)
Active localizers	
Motion-sensitive response	aFM(Mo + FM) > aFM(Stat + FM)
FM-sensitive response	aMo(Mo + FM) > aMo(Mo + nonFM)

General effects of selective attention were also explored beyond the boundary of the auditory cortex. In the absence of *a priori* anatomical predictions, activated regions are reported at a cluster-level threshold of *P* < 0.05, with FDR corrected for whole-brain multiple comparisons to reduce type II errors. Volumetric localization was informed by looking up the probability of the three peaks of maximum significance in each cluster using the corresponding toolbox for spm2 (Eickhoff *et al.*, 2005).

## Results

### Target-detection accuracy

Attention was directed to either the FM of the complex tones, or the movement of the tones in azimuth, by requiring participants to detect oddball instances of spatial movement or FM depth, respectively. To enable direct comparison with the results reported by Krumbholz *et al.* (2007), performance accuracy was transformed into a percentage measure of the hit rate minus FA rate. Hit rate was defined as the number of correct responses divided by the total number of targets, and FA rate was defined as the number of false alarms divided by the total number of false alarms and correct rejections. Table 1 reports the individual performance of the 16 subjects who progressed to the fMRI phase of the experiment. Performance measures were subjected to repeated-measures anova to test for differences across conditions. Target-detection accuracy did not significantly differ between the four attention-demanding tasks during the fMRI experiment (*F*_3,45_ = 2.06, *P* = 0.11).

### Motion and FM sensitivity defined using the passive localizers

**Fig. 2:**
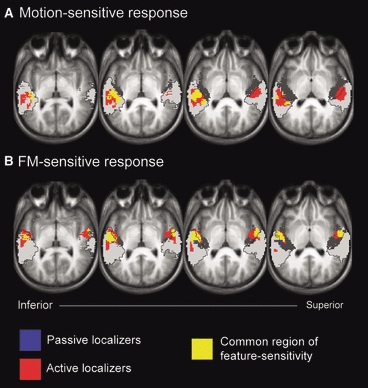
Auditory cortical responses to (A) spatial motion and (B) FM. All clusters survived correction for multiple comparisons (*P* < 0.05). Blue: localizer contrast with passive listening conditions [i.e. A, p(Mo + FM) > p(Stat + FM); B, p(Mo + FM) > p(Mo + nonFM)]. Red: localizer contrast with active listening conditions [i.e. A, aFM(Mo + FM) > aFM(Stat + FM); B, aMo(Mo + FM) > aMo(Mo + nonFM)]. Yellow: overlap between the two contrasts. Activity is overlaid onto the mean normalized anatomical scan for the group. Three oblique axial views are shown. These have been spaced at 3-mm intervals and angled across the supratemporal plane to best illustrate the spatial arrangement of activity with respect to key anatomical sites. The location of Heschl's gyrus (primary auditory cortex) is indicated by the underlying dark grey shading, and the location of the planum temporale and planum polare (non-primary auditory cortex) is indicated by the underlying light grey shading.

Bilateral auditory cortical activity was observed in response to motion and FM (Fig. 2). These activation clusters formed the primary ‘localizer’ regions of interest, in which we then quantified the modulatory effects of selective attention. Note that these regions were not defined with the use of an experimental condition that was a component of the subsequent selective attention analysis.

Motion sensitivity was positioned behind Heschl's gyrus (Fig. 2A, blue and yellow). The peaks of activity occurred in the anteromedial part of the planum temporale at *x* = −52, *y* = −22, and *z* = 4 mm, and *x* = 52, *y* = −22, and *z* = 8 mm, in the left and right hemispheres, respectively. The motion localizer contained 368 voxels on the left. Note that activation on the right was too small to survive the corrected threshold for cluster-level significance (*P* = 0.06, 62 voxels).

FM sensitivity was mostly centred on Heschl's gyrus (Fig. 2B, blue and yellow). The peaks of activity were situated in the lateral part of Heschl's gyrus at *x* = −54, *y* = −10 and *z* = 0 mm on the left [Temporal (Te) 1.2, 80% localization confidence] and in the most anterolateral part of the planum temporale at *x* = 60, *y* = −6 and *z* = −2 mm on the right. The FM localizer contained 472 voxels on the left side and 213 voxels on the right side.

### Region-of-interest analysis for the effect of selective attention

To quantify the effect of attention on the auditory cortical response to the behaviourally relevant stimulus feature, we computed the mean effect size within the FM-sensitive and motion-sensitive regions defined by the passive localizer. Specifically, we used the regions of interest for each participant to compute the mean value of the beta weights for each experimental condition estimated from the general linear model. This metric provides an estimate of the magnitude of the response.

For the FM regions, individual parameter estimates were subjected to a 7 × 2 repeated-measures anova with condition and hemisphere as factors. A one-way anova was performed for the left-sided response to stimulus motion. In both cases, there was a significant effect of condition (motion, *F*_6,90_ = 19.33, *P* < 0.001; FM, *F*_6,90_ = 22.34, *P* < 0.001), and sphericity assumptions were valid. There was no main effect of hemisphere for FM (*F*_1,15_ = 3.09, *P* = 0.10), or any interaction between condition and hemisphere, and so hemispheres were collapsed for further comparisons.

**Fig. 3:**
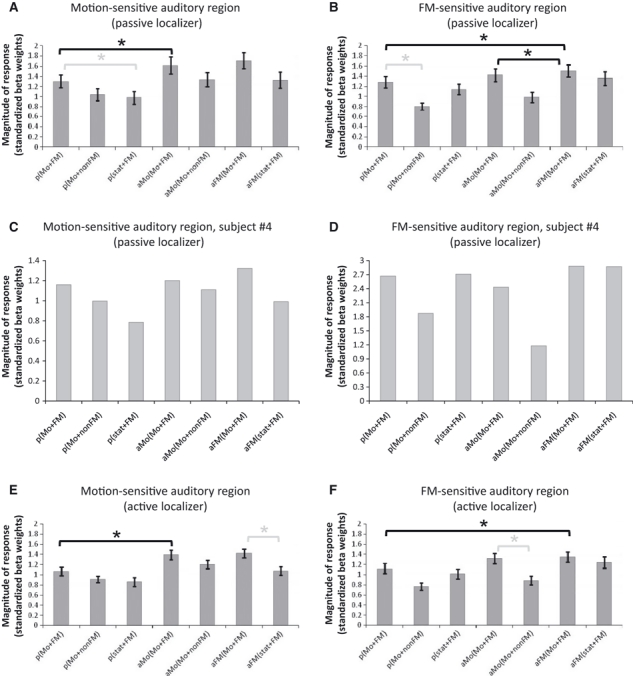
Estimate of the response magnitude for all seven experimental conditions, in motion-sensitive (left panels) and FM-sensitive (right panels) regions. Note that C and D show the results for a single participant (no. 04), whereas the rest of the panels (A, B, E and F) show group data. Finally, the two panels at the bottom depict results for the active localizers, and the rest of the panels show the results for the passive localizers. The error bars denote ± 1 standard error of the mean. Mean data are collapsed across hemisphere. The lines and stars within the panels denote the significant contrasts (grey for the localizer contrasts and black for the attention-related contrasts). * denotes planned pairwise contrasts which reach statistical significance (*P* < 0.05).

Given that the anova is an omnibus statistical test, we explored the pattern of condition-specific differences according to our hypotheses. The size of the response for each of the seven sound conditions is shown in Fig. 3A and B. The crucial directional planned comparison of interest examined the effect of selective attention in enhancing the response in a feature-specific manner. In the FM-sensitive region, this effect [aFM(Mo + FM) > aMo(Mo + FM)] was small, but significant (*t*_31_ = −2.23, *P* = 0.02). In the motion-sensitive region, the corresponding effect [aMo(Mo + FM) > aFM(Mo + FM)] did not reach significance (*t*_15_ = −1.30, *P* = 0.11).

Further analysis explored how consistently these patterns were shown by individual participants. Figure 3C and D displays data from one subject (no. 04), using the same format as in Fig. 3A and B. For the FM-sensitive region (Fig. 3D), attending to FM elicited greater activity than attending to motion [aFM(Mo + FM) > aMo(Mo + FM)] in 11 of 16 subjects. Hence, the group significance reflected consistency across subjects. Figure 3C shows single-subject activation for the motion-sensitive region. Like subject no. 04, a several other subjects in our sample (six of 16) showed enhancement as a result of attending to motion [aMo(Mo + FM) > aFM(Mo + FM)].

Although it partly supports the claim for feature-specific attentional enhancement within the human auditory cortex, the present finding is somewhat in contrast to the evidence reported previously (Ahveninen *et al.*, 2006; Krumbholz *et al.*, 2007; Altmann *et al.*, 2008). For the present dataset, attending to FM enhanced activity in the FM-sensitive region, but attending to spatial motion did not enhance activity in the motion-sensitive region.

We are confident that the lack of consistency in the effect of attending to motion is not simply attributable to the motion localizer being somehow ‘inappropriate’ for some individuals. Considering the magnitude of the responses for those conditions in the passive localizer contrast [p(Mo + FM) > p(Stat + FM); Table 2], individual responses confirmed that the localizer successfully identified voxels sensitive to motion in 15 of 16 subjects. Moreover, in all 16 subjects, this region still preferentially responded to motion under active listening conditions [aFM(Mo + FM) > aFM(Stat + FM)]. Thus, trends across individual subjects support a likely effect of attending to FM in a feature-specific manner in the human auditory cortex, but not of attending to motion.

General effects of attention on auditory cortical responses were also considered. Subsidiary pairwise comparisons at the group level confirmed that, no matter what the focus of attention or the region of interest, target detection significantly increased activity relative to passive listening (for the Mo + FM stimulus) (*P* < 0.01). These effects survived a Bonferroni-corrected threshold for significance (*P* = 0.025). The individual trends confirmed this pattern. In the FM-sensitive regions, activity was also often greater in the active conditions than in the passive conditions [aFM(Mo + FM) > p(Mo + FM), 13 of 16 subjects; aMo(Mo + FM) > p(Mo + FM), 11 of 16 subjects]. Similarly, in the motion-sensitive region there was often an increase in active conditions relative to passive conditions [aFM(Mo + FM) > p(Mo + FM), 13 of 16 subjects; aMo(Mo + FM) > p(Mo + FM), 15 of 16 subjects]. This result indicates that, when participants are actively engaged in a listening task, there are widespread increases in auditory cortical activity, in agreement with results from previous studies (Grady *et al.*, 1997; Hall *et al.*, 2000; Brechmann & Scheich, 2005; Johnson & Zatorre, 2005). The functional interpretation of this finding is less clear, because decision and sensorimotor processes are also uniquely engaged by the attentive listening condition. Evidence indicates that the representation of a sound stimulus in the auditory cortex is actively modulated by the conceptual and executional aspects of the task (e.g. Scheich *et al.*, 2007).

### Comparison between passive and active localizers

Broadly speaking, the active localizers were co-localized to the passive localizers, but were more extensive (Fig. 2). The active localizer for motion contained 1031 voxels on the left and 421 voxels on the right. The active localizer for FM contained 671 voxels on the left and 455 voxels on the right. Each region was thus, on average, approximately two-thirds larger with the active localizers. In this section, we compare these two sets of findings (passive vs. active localizers), so that we might understand the above results in the context of those previous fMRI studies that had defined regions with the use of an experimental condition that was a component of the subsequent selective attention analysis. From these larger regions of interest, the mean parameter estimates were, again, subjected to a 7 × 2 repeated-measures anova, with condition and hemisphere as factors. In both cases, there was a significant effect of task (motion, *F*_2.9,43.5_ = 18.33, *P* < 0.001; FM, *F*_6,90_ = 27.83, *P* < 0.001). There were no main effects of hemisphere. Sphericity assumptions were invalid for motion, and so a Greenhouse–Geisser correction was applied.

Figure 3E and F illustrates the response magnitude across the different conditions when the active localizer was used. Qualitatively, these appear very similar to those obtained with the passive localizer (Fig. 3A and B). However, for the active localizer, neither of the corresponding directional planned comparisons [i.e. aMo(Mo + FM) > aFM(Mo + FM) and aFM(Mo + FM) > aMo(Mo + FM)] reached significance (*P* = 0.26 and *P* = 0.22, respectively). Hence, this null result would appear to no longer support our previous conclusion that selective attention to frequency-modulated targets enhances activity in FM-sensitive regions in the human auditory cortex. In contrast, the general effects of attention were more robust to the choice of localizer. Subsidiary pairwise comparisons confirmed that the two attention-demanding conditions were both significantly greater than passive listening to the same stimulus (Mo + FM) (*P* < 0.001). Again, these effects survived a Bonferroni-corrected threshold for significance (*P* = 0.025).

### Whole-brain analysis for the effect of selective attention

So far, our analyses have exclusively assessed the effects of auditory selective attention within predefined regions of the auditory cortex. In this section, we describe the effects of enhancement by selective attention elsewhere in the brain. This analysis considers whether there might be a network of brain centres engaged by spatial attention, or similarly by nonspatial attention. The contrasts reported are aMo(Mo + FM) > aFM(Mo + FM) and aFM(Mo + FM) > aMo(Mo + FM) (Table 2).

**Fig. 4:**
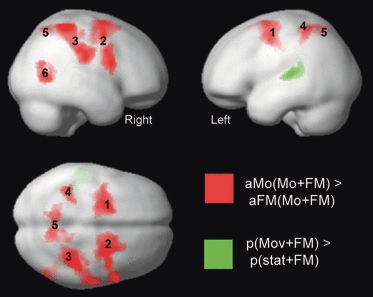
Scope of the network of brain regions engaged when participants are attending to stimulus motion as compared with attending to FM (red). In both conditions, the stimulus is the same. The locations of the differential activity are contrasted with the position of the passive motion localizer (green). This figure clearly illustrates how the motion-related task differences engage higher-level regions, whereas the motion-sensitive response is restricted to the auditory cortex.

Only the former contrast revealed significant activity (Table 3). Attending to the spatial targets increased activity in a widespread number of brain regions, as compared with attending to the frequency-modulated targets in the same stimulus. Figure 4 illustrates how differential activity was primarily centred in the dorsolateral prefrontal cortex (regions 1 and 2) and the postcentral gyrus (regions 3 and 4), with small additional regions of activity around the precuneus (region 5) and the right posterior middle temporal gyrus (region 6). This distribution is in broad agreement with Krumbholz *et al.* (2007), with dorsolateral prefrontal cortex activity also being reported by Degerman *et al.* (2006). Previously, these regions have been considered to control the focus of attention and to filter out stimulus features irrelevant to the task. In the present study, this hypothesis is perhaps less plausible, given our careful matching for perceptual difficulty across spatial and nonspatial tasks. An alternative view might be that the spatial task engages processes additional to those engaged by the nonspatial task. For example, mentally updating spatial information (spatial working memory) might engage the lateral superior frontal gyrus (regions 1 and 2) (Tanaka *et al.*, 2005), whereas imagining the corresponding visual analogues of auditory motion might reasonably involve the precuneus (region 5) (Culham *et al.*, 1998; Hanakawa *et al.*, 2003).

**Table 3:** The location and extent of the specific enhancement during attention to spatial motion as compared with attention to FM [aMo(Mo + FM) > aFM(Mo + FM)], with a cluster-level threshold of *P* < 0.05, and controlled for multiple comparisons by use of the FDR; coordinates and *Z*-values are reported for the three peaks of maximum significance within each cluster

Peak MNI coordinate (mm)							
					
*x*	*y*	*z*	Cluster size (no. of voxels)	*Z*-value	Side	Putative anatomical region	Label on Fig. 4
−18	0	62	1139	5.37	Left	Superior frontal gyrus	1
−30	−6	46		4.57	Left	Precentral gyrus	1
−28	0	50		4.53	Left	Middle frontal gyrus	1
30	−8	58	1829	4.72	Right	Superior frontal gyrus	2
40	2	52		4.54	Right	Middle frontal gyrus	2
56	8	20		4.39	Right	Inferior frontal gyrus	2
38	−44	60	1603	5.23	Right	Postcentral gyrus	3
62	−20	28		5.12	Right	Supramarginal gyrus	3
52	−28	36		4.53	Right	Supramarginal gyrus	3
−40	−44	54	294	4.26	Left	Postcentral gyrus	4
−26	−36	58		3.85	Left	Postcentral gyrus	4
−28	−42	46		3.48	Left	Postcentral gyrus	4
10	−60	56	553	4.11	Right	Precuneus	5
−10	−56	62		4.00	Left	Precuneus	5
16	−44	58		3.75	Right	Postcentral gyrus	5
52	−62	6	387	4.42	Right	Middle temporal gyrus	6
54	−70	16		3.69	Right	Middle temporal gyrus	6
56	−68	0		3.41	Right	Middle temporal gyrus	6

## Discussion

### Summary of results

This fMRI study sought evidence for attentional modulation in feature-sensitive regions of the human auditory cortex when the attention-demanding tasks had been matched for difficulty during a pre-scanning assessment. The location of FM-sensitive and motion-sensitive responses was broadly compatible with previous research, and involved the lateral Heschl's gyrus and planum temporale, respectively. Within these regions, we examined evidence for attentional enhancement during attention to spatial (auditory motion) and nonspatial (FM) features of the stimulus. The results revealed the novel finding of response enhancement by selective attention for the nonspatial (FM) feature, but not for the spatial (motion) feature. Instead, attending to spatial motion produced relative increases in activity elsewhere in the brain, in particular the dorsolateral prefrontal cortex and postcentral gyrus. Activity in these regions might possibly be associated with additional decision and sensorimotor demands of the spatial listening task, but the present data cannot confirm precisely what these functions might be. The results also confirmed reliable enhancement of the response within auditory regions during attention to either feature, as compared with passive listening (see also Ahveninen *et al.*, 2006; Paltoglou *et al.*, 2009). Here, we consider the implications of these findings.

### Feature-specific modulation in the auditory cortex

Using the passive localizer, our primary analysis considered auditory cortical regions in which the stimulus contrast was independent from the contrasts used to examine the differential effects of the focus of attention. The results demonstrated evidence for attention-related, feature-specific enhancement during attention to frequency-modulated targets as compared with attention to motion targets occurring within the same stimulus sequence. Thus, our results, in part, support the hypothesis that, as in the visual system, attention enhances the representation of an attended nonspatial feature within the corresponding sensory region. Our positive finding of an effect of attention to a nonspatial feature is perhaps most simply interpreted as being related to our choice of FM as the nonspatial feature of interest, because the use of other nonspatial features, such as phoneme identity and pitch, has resulted in null findings (Ahveninen *et al.*, 2006; Altmann *et al.*, 2008; Krumbholz *et al.*, 2007). The choice of FM was motivated by previous reports of strong, sustained cortical activation in response to slow-rate FM within a single region of the human auditory cortex. Specifically, slow-rate FM (< 5 Hz) elicits focal activity around the lateral portion of Heschl's gyrus and the lateral planum temporale, which is in agreement with the results of our study (Griffiths *et al.*, 1998; Binder *et al.*, 2000; Thivard *et al.*, 2000; Brechmann *et al.*, 2002; Hall *et al.*, 2002; Hart *et al.*, 2004; Brechmann & Scheich, 2005).

To our surprise, the region-of-interest analysis did not support previous findings of enhancement during attention to the spatial features of a stimulus (see Ahveninen *et al.*, 2006; Altmann *et al.*, 2009; Krumbholz *et al.*, 2007). This form of enhancement may be quite sensitive to factors other than attention, such as experimental design or choice of region of interest. The lack of an effect of spatial attention is particularly intriguing, given our ability to reliably identify the sensory response to spatial motion. Krumbholz *et al.* (2007) suggested that a task must be fairly difficult in order for reliable attention-related modulations in activation to be detected. Insufficient task difficulty is unlikely to account for the present result, because detection accuracy did not reach the ceiling for either the aMO(Mo + FM) or aFM(Mo + FM) conditions. Furthermore, although performance in the MR scanner was not as closely matched as it had been during the pre-scanning assessment, the mean inter-subject difference in performance was still quite good (6.6%; Table 1). Perhaps neural operations associated with spatial attention were distributed across the higher-level brain regions shown in Fig. 4.

Two further issues are worthy of consideration. First, we acknowledge the ongoing debate about whether auditory attention operates more effectively at a representational level, in which features are conjoined and bound to an auditory object, than at the level of individual feature coding (Cusack & Carlyon, 2003; Shamma, 2008). According to this argument, attending to spatial properties might not only modulate activity in auditory regions that process motion, but also influence activity in FM-processing regions, because both features belong to the same object. Parsimony, perhaps, does not favour this argument, because its logic fails to account for the internal discrepancy with our positive effect of attending to FM, and also with previous fMRI studies. The second explanation is related to our previous account of the positive effect of attending to a nonspatial feature and follows thus. In an experimental design in which the main contrast is determined by manipulating the focus of attention to one of two different features belonging to the same sound stimulus, it is possible that the effect of attention is crucially dependent on the choice of the nonspatial feature of interest. According to this argument, enhancement during attention to spatial features might be evident when compared with attention to pitch or phonemes, but not FM. In Fig. 3A, there is some trend (although not significant) for attention to FM to generally increase activation, even in the motion-sensitive region. Thus, one possible explanation for the lack of effect of selective attention to the spatial cue is that attending to FM slightly increases auditory cortical activity even in a region that responds more to spatial motion under passive listening conditions. Although this proposal is speculative at present, this argument has some empirical support elsewhere. Notably, a series of behavioural studies of auditory selective attention have shown how auditory discrimination is not equivalent for all features, and that the discriminability of the target depends on its relationship with the distractors (Cusack & Carlyon, 2003).

### Comparing passive and active localizers

The present experimental design permitted us to explore the way in which the listening task might influence the location and extent of the FM-related and motion-related sensory activation. In practice, there is little consensus regarding the way in which feature-sensitive activity has been localized. Evidence from electrophysiological recordings from awake-behaving macaques supports the use of a passive behavioural state in providing a valid representation for localizing sensory activity, and indicates that a painstaking procedure of training the animals to perform a task as a control for selective attention is not necessarily more advantageous (Scott *et al.*, 2007). Some studies on humans have attempted to control the localizer contrasts by directing the participant's response either to visual stimuli (Petkov *et al.*, 2004) or to an irrelevant feature of the same stimulus (Krumbholz *et al.*, 2007), whereas others prefer passive listening (Paltoglou *et al.*, 2009) or instruct participants to ignore the sounds (Ahveninen *et al.*, 2006).

The present results demonstrated that when control was exerted over the listening context by requiring participants to attend to the irrelevant feature, the feature-related response was rather more widespread than with the corresponding passive localizer. In fact, the passive localizer was almost a subset of the active one. The two contrasts both appeared to be successful in localizing sensory activation, although the passive contrast failed to produce a significant cluster of motion-sensitive activation in the right hemisphere.

Although passive and active localizers appeared to be qualitatively similar, a significant increase in response magnitude in the FM-sensitive region as a function of attending to FM was found only when the passive localizer was used. This is an unexpected result, considering the argument advanced previously (Krumbholz *et al.*, 2007) that a localizer based on active listening is better controlled than one based on passive listening. It might also be considered to be unforeseen, because the active localizer includes more voxels, and so should yield a mean response that is less susceptible to noise. Our findings suggest quite the opposite; the apparent reduction in sensitivity implies that attentional modulation is restricted to those voxels identified by the passive localizer. The reduction in sensitivity probably also reflects correlations introduced between the localizer and the effect of selective attention by use of the same stimulus condition in both contrasts (Kriegeskorte *et al.*, 2009). To illustrate this point, let us consider the analysis of the FM-sensitive region. The FM active localizer was defined by comparing the conditions aMo(Mo + FM) and aMo(Mo + nonFM), such that both conditions required attention to the irrelevant feature, with the only difference being the presence of FM (similar to Krumbholz *et al.*, 2007). In this contrast, the response to aMo(Mo + FM) must be significantly greater than to the other condition. The effect of selective attention to FM was subsequently defined by comparing the conditions aFM(Mo + FM) and aMo(Mo + FM) for those voxels identified by the localizer. In this contrast, the response to aMo(Mo + FM) must be significantly smaller than that to the other condition. Thus, the response to aMo(Mo + FM) determines both the sensory response and the modulatory effect of attention, but its relation to the other condition must be in the opposite direction. Taking the hypothetical case where measurement noise (with a zero mean) is added to voxel responses in the aMo(Mo + FM) condition, the result is the addition of some voxels to the localizer and the removal of others. The voxels added would be those with a greater response to aMo(Mo + FM) than to aMo(Stat + FM). In subsequent contrasts, these voxels would be more likely to show a greater response to aMo(Mo + FM) than to aFM(Mo + FM). Hence, the correlation introduced between the localizer and the effect of selective attention effectively sets up a bias towards a reduced effect size for the contrast of interest. Although we cannot be completely sure of the reason why the effect disappears for the active localizer, this is a persuasive theoretical argument in favour of using a passive localizer, or at least one generated by an independent set of experimental conditions. Correlations can be viewed as flaws in experimental design that would generate false-positive responses, and could equally obscure an effect of interest.

## Abbreviations

FDR: false discovery rate
FM: frequency modulation
fMRI: functional magnetic resonance imaging
ITD: interaural time difference
Te: temporal
